# Are sex and gender considered in head and neck cancer clinical studies?

**DOI:** 10.1038/s41698-023-00439-z

**Published:** 2023-09-07

**Authors:** Aurora Gaeta, Marta Tagliabue, Oriana D’Ecclesiis, Lavinia Ghiani, Paolo Maugeri, Rita De Berardinis, Camilla Veneri, Camilla Gaiaschi, Marina Cacace, Luciano D’Andrea, Mohssen Ansarin, Sara Gandini, Susanna Chiocca

**Affiliations:** 1 Department of Experimental Oncology, IEO, European Institute of Oncology IRCCS, Via Adamello 16, 20139 Milan, Italy; 2 Department of Otolaryngology Head & Neck Surgery, IEO, European Institute of Oncology IRCCS, 20141 Milan, Italy; 3 Department of Biomedical Sciences, University of Sassari, Sassari, Italy; 4 GENDERS (Gender & Equality in Research and Science) – University of Milan, Milan, Italy; 5 Knowledge & Innovation, Via Guido Reni 56, Rome, 00196 Italy; 6 Present Address: Department of Human and Social Sciences, University of Salento, Lecce, Italy

**Keywords:** Head and neck cancer, Oral cancer

## Abstract

We analyzed the inclusion of sex and/or gender (S/G) in Head and Neck Cancer (HNC) clinical studies, through inspecting ClinicalTrials.gov (AACT) and the mention of Human Papilloma Virus (HPV) on a specific subgroup, namely oral cavity, larynx and oropharynx. Only 5% of HNC studies mention S/G as a planned analytical variable. Proportionally more observational studies treated S/G as an analytical variable than interventional studies (10% vs 5%, *P*-value ≤ 0.001), 8% of studies that mentioned S/G involved more than 100 subjects while 4% less than 100 (*P*-value ≤ 0.001). In randomized protocols, S/G was mentioned more in studies with a planned sample of more than 100 patients and including HPV status (*P*-value < 0.05). Small controlled studies have lower mention of S/G as an analytical variable than uncontrolled studies (4% and 10%, respectively among studies with less than 100 subjects). Significantly greater mention of S/G as an analytical variable is observed in controlled and randomized studies with a sample size greater than 100 subjects. HPV was mentioned in only 18% of oral cavity-larynx-oropharynx studies. Interventional studies do not regularly account for S/G during HNC study design. Thus, although fundamental, in studies concerning HNC the S/G variable is often not considered. In trials published in scientific journals (*P*-value = 0.01) and in more recent clinical trials (*P*-value = 0.002), S/G is taken more into account suggesting an increasing awareness on its importance. However, the need to systematically include S/G in study design clearly emerges, to better highlight sex-related differences in disease incidence and prognosis and best imbue science and medicine with the proper biological and cultural differences.

## Introduction

Sex and/or gender (S/G) differences among individuals have an impact on health, an issue more developed in other areas of medicine than in oncology. The two terms are often used interchangeably, but it must be emphasized that they should not be used synonymously, especially when designing clinical trials^1–4^. Sex refers to biological characteristics and should be analyzed in humans, animals, organs, cells, and their components. Gender refers to self-identification and includes the socially constructed roles, expectations, relationships, behaviors, and other traits that societies ascribe to women, men and people of diverse identities. Importantly, sex and gender have a complex relationship as they continuously interact during an individual lifetime^5^. Although the relevant difference between these terms is commonly known, they are not always carefully distinguished in clinical studies. Therefore in this study, we focused on the inclusion of general terms that refer to sex and gender (S/G) without distinction, in head and neck cancer (HNC) clinical studies, as described in the methods section. This strategy, although sub-optimal, as it does not tease out the two terms properly, allows to use sex as a proxy for gender, particularly when the former is connected to other risk factors emerging from lifestyle and behavior^6^. In this respect, this study can be considered as a snapshot of the current state of the art of how sex and gender analysis is currently carried out in HNC clinical studies. Sex differences have been extensively reported in many cellular and molecular pathways relevant to cancer onset^3^. In HNC, disparities by sex and race/ethnicity were first discussed in a landmark report by Blot et al.^7^.

HNC is a highly heterogeneous group of tumors arising in the epithelial cells of mucosal linings of different anatomical sites of the head and neck district. HNC is more frequent in men than in women, with an incidence ratio approximately equal to 3:1 and is generally diagnosed at an average age of 50-70 years^8–12^. HPV contribution to HNC is substantial but highly heterogeneous by cancer site, continent/region, and sex. In the last decades, high-risk Human Papillomavirus (hr-HPV, especially HPV16) infection has been established as associated to a subset of oropharyngeal cancers^13–15^. Patients with HPV-positive HNC present a better prognosis, including a lower risk of local disease recurrence than HPV-negative HNC patients^10,16,17^. The epidemiology of the infection was observed in a study conducted in the United States: Men had a significantly higher prevalence than women for any genotype oral HPV infection (10.1% [95% CI, 8.3-12.3] vs 3.6% [95% CI, 2.6-5.0], *P*-value < 0.001). Most of the studies conducted among healthy individuals showed that oral HPV16 prevalence is low, between 0.5-1%, and was consistently lower than ano-genital HPV16 prevalence for both men and women^18^. Currently, while racial inequalities in oral cavity cancer incidence have declined in recent years, sex disparities are still persisting, occurring approximately two to three times more frequently in men^19^, and remain mostly unexplained^20^. Studies have implicated the differential expression patterns of sex hormone receptors in HNC, but with unclear significance, which is not surprising giving the heterogeneity of these tumors^21^. In oropharyngeal cancers, where most HPV-positive cancers occur, racial/ethnic and sex disparities in incidence have amplified in recent years, with a white male predominance today. Indeed, it is one of the cancers with the fastest rise in incidence in United States non-Hispanic white men^13^. This notion must be viewed within the context of findings describing that women are both under-represented in clinical trials and under-treated^22–25^, and also that women with HNC may be more financially fragile compared to men affected by the disease^26^. All Phase III clinical trials must include subgroup analyses to assess gender as well as racial/ethnic differences in treatment efficacy, even when small sample size may limit statistical power^27^, to fully evaluate S/G differences in all phases of oncology, including the ones described in our study. Similarly, it is therefore important in HNC studies to take into account gender-specific medicine, which aims to study how S/G variables influence disease progression. Given the rarity of oral HPV infection, large sample sizes are needed to both assess the natural history of oral HPV and score significant differences among S/G. Our analysis follows the path traced by Brady et al.^28^ “*Lack of consideration of sex and gender in COVID-19 clinical studies*”, focusing on the mention of S/G in clinical studies concerning HNC. A large amount of information is available by ClinicalTrial.gov through AACT and, depending on the objectives of the study, an assessment of the information to be extracted is necessary. To verify the inclusion of all studies registered in the National Clinical Trial system, a systematic review was also conducted on PubMed and Embase.

Clinical trials in oncology have the greatest female underrepresentation relative to the female proportion of corresponding Disability-adjusted life year (DALYs)^29^. It is crucial to look at the role of S/G in interventional or randomized studies, especially since upon comparison to their respective disease prevalence, female participants are more underrepresented^25^. Biases in clinical trials have been identified as the primary scientific and methodological error since the early days of the debate on gender medicine, leading to the construction of the male standard patient in medicine^30–33^. Until recently, United States and Canada were the only countries having explicit requirements to include women in clinical trials and to perform sex-based subgroup analysis on study results^34^. Nowadays, including both sexes at an earlier stage of the experiment is a well understood practice within the scientific and medical community^35^. By comparing a recent bibliometric analysis conducted in 2019 to the results of a 2009 similar study, a significant increase in the studies including both sexes in all nine medical disciplines is apparent^36^. In 2019 49% of the studies included both sexes, against 28% in 2009^37^. Here we set to identify the awareness and attention to S/G at the registration and publication phase in HNC clinical studies. A validation step was further conducted by relating our findings on the trials registered in Clinicaltrial.gov to the corresponding peer-reviewed published studies. Finally, we compared S/G consideration to HPV status reporting, an important risk factor in HNC with a recent rising trend.

## Results

### HNC studies selection

**Fig. 1:**
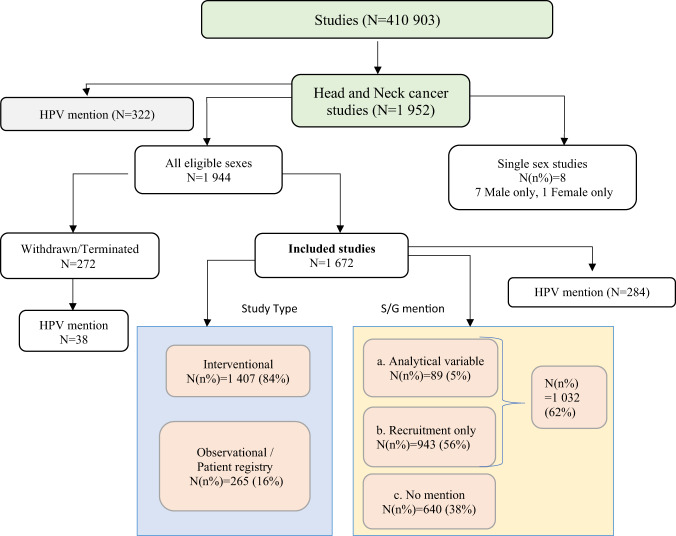
Distribution of head and neck cancer studies by type of Sex and Gender (S/G) mention and by study type. 1952 studies were selected (from 410 903 studies present in date 12/04/2022), 272 were excluded because they were withdrawn or terminated and 7 + 1 = 8 studies were excluded because presented exclusively male and female as eligible sexes, respectively. The left panel shows the distribution of the 1672 identified studies across study types (the classifications are taken from ClinicalTrials.gov). The right panel shows the distribution of studies in the various mutually exclusive S/G groups that we defined (see Methods section). There is a hierarchy in the ‘Some S/G mention’ set. The majority of studies mentioned S/G; among these, studies that only mentioned it in the recruitment criteria predominated.

**Fig. 2:**
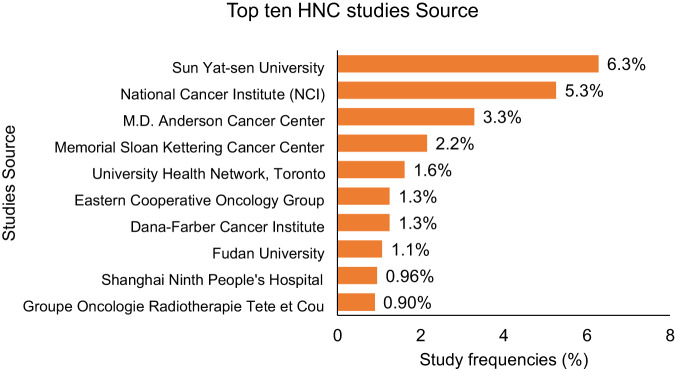
Top 10 most frequent publication source. Percentage of Head and Neck Cancer (HNC) Top 10 most frequent publication source of the 1672 identified studies included on ClinicalTrials.gov (from 1999-to 12/04/2022).

We identified 1952 HNC studies registered on ClinicalTrials.gov (Fig. 1). Two hundred and eighty studies were not included in the analysis: 272 were withdrawn or terminated while 8 studies enrolled either males only (7 studies) or females only (1 study). The main sources of studies regarding HNC on ClinicalTrials.gov were the Sun Yat-Sen University (Fig. 2) which was in charge of 6.3% of the selected studies, followed by *National Cancer Institute NCI*, in charge of 5.3% of the selected studies and *M.D. Anderson Cancer Center* (3.3%) (Fig. 2). We also conducted a systematic review (Supplementary Fig. 1 and Supplementary Fig. 2, see Methods).

### S/G mention in HNC studies

**Fig. 3:**
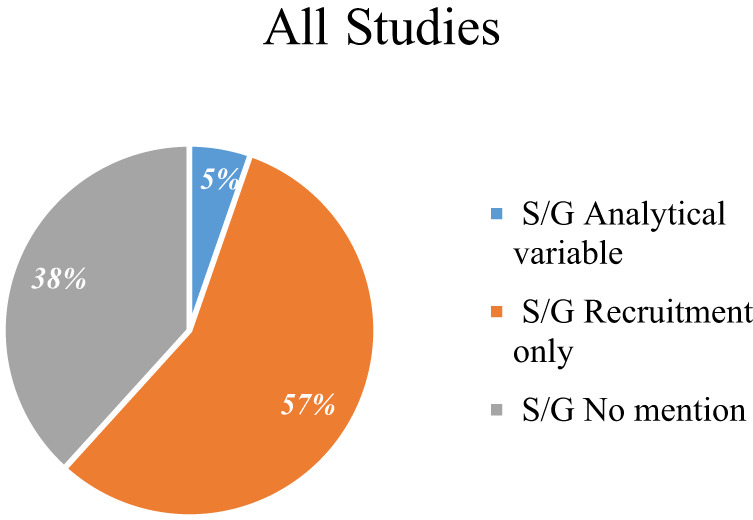
Sex/gender mentioned in all studies reported in Table 1. The S/G variable is mentioned in the studies as a recruitment factor, as an analytical variable or not considered (No mention).

Out of the 1672 studies selected, 1032 (62%) explicitly made some S/G mention in the sections targeted by our search (see Methods section); of those, 943 (56%) explicitly addressed S/G solely as a recruitment criterion and only 89 (5%) mentioned S/G as a planned analytical variable (Table 1, Supplementary Table 1- 1/a). Figure 3 refers to the S/G mentioned in all studies reported in the main analysis. Among the studies analyzed, the randomized studies were 538 (32%), while 1134 (68%) were not randomized (Supplementary Table 2). Two-hundred and sixty-five (16%) were observational/patient registry studies and 1407 (84%) were interventional (Table 1). There was a significant difference in the S/G role addressed in different types of studies (*P*-value < 0.001). In interventional studies the use of S/G only as a recruitment criterion prevailed (64%), whereas in observational/patient registry studies the absence of its mention predominated (73%). Proportionally more observational/patient registry studies treated S/G as an analytical variable than interventional studies (10% vs 5%). The absence of mention prevailed in observational studies (73%) compared to interventional studies (32%) (Table 1). Significant differences were observed between mention of S/G and the overall status of the study (*P*-value = 0.002). In particular, the majority of studies included were completed (43%), while 24% were in the recruitment phase or enrolling by invitation. Overall, the mention of S/G prevailed as a recruitment criterion only. We observed that active but not recruiting studies, together with studies suspended or with unknown status, mentioned more S/G as an analytical variable (7% both, *P*-value = 0.002) (Table 1).

**Table 1:** Studies characteristics by Sex/Gender mention, coded as described in Methods; data were collected on 12 April 2022.

Variable	Overall *N* = 1672^a^	Analytical variable *N* = 89^a^	Recruitment only *N* = 943^a^	No mention *N* = 640^a^	*P*-value^b^
*Study type, (n%)*					<0.001
Interventional	1407 (84)	63 (4.5)	897 (64)	447 (32)	
Observational /Observational [Patient Registry]	265 (16)	26 (9.8)	46 (17)	193 (73)	
*Overall status, (n%)*					0.002
Active, not recruiting	174 (10)	12 (6.9)	113 (65)	49 (28)	
Completed	724 (43)	33 (4.6)	382 (53)	309 (43)	
Not yet recruiting	108 (7)	6 (5.6)	68 (63)	34 (31)	
Recruiting/Enrolling by invitation	396 (24)	20 (5.1)	244 (62)	132 (33)	
Unknown status/Suspended	270 (16)	18 (6.7)	136 (50)	116 (43)	
*Enrolment, (n%)*					<0.001
≤100^c^	1181 (70,5)	51 (4.3)	699 (59)	431 (36,5)	
>100	491 (29,5)	38 (7.7)	244 (50)	209 (43)	
*HPV, (n%)*					0.075
Mention	284 (17)	18 (6.3)	174 (61)	92 (32)	
No mention	1388 (83)	71 (5.1)	769 (55)	548 (39)	
*Studies with a relevant HPV role (n%)*					0.030
Oral cavity, oropharynx and larynx subsites	565 (34)	40 (7.1)	325 (58)	200 (35)	
Others	1107 (66)	49 (4.4)	618 (56)	440 (40)	
Phase, (*n*%)	1407 (84)	63 (4.5)	897 (64)	447 (32)	<0.001
Early Phase1/Phase1	182 (13)	7 (3.8)	142 (78)	33 (18)	
Phase 1/Phase 2	101 (7)	4 (4)	69 (68)	28 (28)	
Phase 2/Phase 3	820 (58)	33 (4)	573 (70)	214 (26)	
Phase 4	17 (1)	0 (0)	(71)	5 (29)	
Not Applicable^c^	287 (20)	19 (6.6)	101 (35)	167 (58)	

Studies were assigned exclusively in one category. Information regarding the overall recruitment status was present in 91% of our set.

^a^*n* (%).

^b^Pearson’s Chi-squared test or Fisher’s test.

^c^Missing values included.

Seventy percent of the studies had a size of less than 100 subjects (*n* 1146) or missing information on sample size (*n* 35). The S/G mention differed significantly according to sample size (namely studies including either more or less than 100 subjects, *P*-value < 0.001). In proportion, 8% of the studies with more than 100 subjects and 4% with less than 100 subjects mentioned S/G as an analytical variable. Studies with less than 100 subjects mentioned S/G only as recruitment criteria to a greater extent when compared to studies with more than 100 subjects (59% vs 50%) (Table 1).

For the interventional studies, information concerning the study phase was provided. Most of the interventional studies were phase 2/phase 3 (58%). We highlighted a significant difference between the S/G role and the different trial phases (*P*-value < 0.001). In particular, we observed a higher percentage of S/G mentioned as an analytical variable in phase 2/3 (4%), early phase 1/phase1 (4%), and where the phase definition was not applicable (6.6%). S/G was specified in the inclusion criteria in 78% of early-phase1/phase 2 trials and 68% of phase1/phase 2 investigations, respectively (Table 1).

In Supplementary Fig. 3 related to Supplementary Table 1-1/a and Table 1, where any mention of S/G is compared, the confidence interval underlines that phase 2/phase 3 studies mention significantly less S/G than the early phase 1/phase 1 studies (OR = 0.63, 95% CI [0.42–0.94]). Studies that quoted HPV mentioned significantly more S/G than those that did not (Supplementary Table 1-1/a). S/G was mentioned significantly more in research with planned sample sizes of under 100 patients as compared to studies with sample sizes over 100 (OR = 1.29 95 CI [1.04–1.60]). Interventional studies cited significantly more S/G in the protocol than observational studies (OR = 5.76 95 CI [4.29–7.72]).

**Fig. 4:**
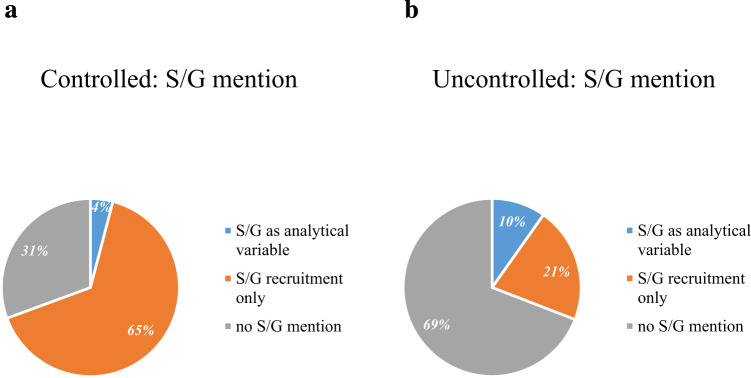
Sex/Gender mention in controlled and uncontrolled studies. The S/G variable is mentioned differently in controlled (**a**) or uncontrolled studies (**b**) as a recruitment factor, as an analytical variable or not considered (no S/G mention).

Figure 4 shows S/G in controlled and uncontrolled studies. Controlled Interventional studies have a significantly higher percentage of S/G mention as recruitment criteria than uncontrolled studies (65% vs 21%, *P*-value < 0.001, Fig. 4, Table 2) and a lower mention of S/G as analytical variable (5% and 10%, respectively).

**Table 2:** Description of characteristics for controlled studies and uncontrolled studies.

Variable	Controlled studies					Uncontrolled studies					P-value^b,c^
	Overall *N* = 1212^a^ (col %)	Analytical variable, *N* = 58^a^	Recruitment only *N* = 785^a^	No mention *N* = 369^a^	*P*-value^b^	Overall, *N* = 204 (col %)	Analytical variable *N* = 20^a^	Recruitment only *N* = 43^a^	No mention *N* = 141^a^	*P*-value^b^	
*Study type (n%)*					-					0.01	-
Interventional	1212	58 (4.8)	785 (65)	369 (30)		52 (25)	4 (7.7)	19 (37)	29 (56)		
Observational/Patient Registry						152 (75)	16 (11)	24 (16)	112 (74)		
*Overall status (n%)*					0.02					0.9	0.07
Active, not recruiting	149 (12)	9 (6)	107 (72)	33 (22)		21 (10)	2 (9.5)	5 (24)	14 (67)		
Completed	481 (40)	19 (4)	291 (60)	171 (36)		81 (40)	9 (11)	16 (20)	56 (69)		
Not yet recruiting	95 (8)	4 (4.2)	66 (69)	25 (26)		9 (4)	1 (11)	2 (22)	6 (67)		
Recruiting/Enrolling by invitation	318 (26)	15 (4.7)	221 (69)	82 (26)		51 (25)	4 (7.8)	13 (25)	34 (67)		
Unknown status/Suspended	169 (14)	11 (6.5)	100 (59)	58 (34)		42 (21)	4 (9.5)	7 (17)	31 (74)		
*Enrolment (n%)*					0.009					0.03	<0.001
≤100^d^	879 (73)	32 (3.6)	573 (65)	274 (31)		116 (56)	12 (10)	32 (27)	72 (63)		
>100	333 (27)	26 (7.8)	212 (64)	95 (29)		88 (43)	8 (9.1)	11 (12)	69 (78)		
HPV mention (*n*%)	222 (18)	13 (5.9)	160 (72)	49 (22)	0.01	43 (21)	3 (7.0)	8 (19)	32 (74)	0.7	0.40
*Phase (n%*)					<0.001					0.05	<0.001
Early Phase 1/Phase1	159 (13)	6 (3.8)	125 (79)	28 (18)		4 (2)	0 (0)	3 (75)	1 (25)		
Phase 1/Phase 2	85 (7)	4 (4.7)	61 (72)	20 (24)		2 (1)	0 (0)	1 (50)	1 (50)		
Phase 2/Phase 3	717 (59)	33 (4.6)	500 (70)	184 (26)		15 (7)	0 (0)	9 (60)	6 (40)		
Phase 4	16 (1)	0 (0)	12 (75)	4 (25)							
Not Applicable^d^	235 (19)	15 (6.4)	87 (37)	133 (57)		183 (90)	20 (11)	30 (16)	133 (73)		

The arms’ description was investigated within the dataset design groups. If at least one arm was defined as “Experimental”, “Active Comparator”, “Placebo Comparator”, “Sham Comparator” the study was classified as controlled, otherwise it was classified as uncontrolled (“No intervention”, “Other”).

^a^*n* (%).

^b^Pearson’s Chi-squared test or Fisher’s test.

^c^P-value from a Pearson’s Chi-squared test or Fisher’s test for controlled versus uncontrolled studies.

^d^missing values included.

Among the uncontrolled studies, whether interventional or observational, the percentage of those not mentioning S/G terms prevailed (56% and 74%, Table 2). Among controlled trials, a statistically higher proportion of studies included less than 100 subjects compared to uncontrolled studies (73% vs 56%, *P*-value ≤0.001). Controlled studies mentioned S/G as an analytical variable significantly more frequently when the study enrolled more than 100 subjects (8% vs 4%, *P*-value = 0.009). In uncontrolled studies, S/G was mentioned significantly more as an enrolment criterion when the sample size declared was lower than 100 patients (27% vs 12%, *P*-value = 0.03). Only 6% and 7% of controlled and uncontrolled studies, respectively, that mentioned S/G as an analytical variable also mentioned HPV (Table 2); no significant difference was observed between HPV mention and controlled study (*P*-value = 0.40, Table 2). In controlled studies, HPV status was significantly more mentioned in studies that quoted S/G as a recruitment criterion (72%, *P*-value = 0.01, Table 2).

Supplementary Table 1-1/b shows the comparison of controlled and uncontrolled studies for any S/G mention (divided as S/G mention (a + b) vs no mention (c)-see methods).

Supplementary Table 2 shows S/G mention in randomized and non-randomized studies. Of the 1672 studies included, 538 were described as randomized.

In randomized trials, studies that dealt with HPV-associated HNC mentioned S/G significantly more at the time of study submission, as reported in ClinicalTrial.gov, than studies with non-HPV-associated diseases (OR not HPV relevant condition vs HPV relevant condition = 0.55, 95% CI [0.38–0.79]). S/G was mentioned in randomized trials with a planned sample of more than 100 patients and in early phase 1 studies versus not applicable (Supplementary Fig. 4).

### HPV mention in HNC studies

HPV is mentioned in 17% of HNC studies including all eligible sexes (Fig. 1). No statistically significant difference between the mention of S/G and the mention of HPV was observed (*P*-value = 0.075, Table 1). Among the 284 (17%) studies that mentioned HPV most of them mentioned S/G in the recruitment (61%), 32% did not mention S/G, and 6.3% reported it as an analytical variable (Table 1).

**Fig. 5:**
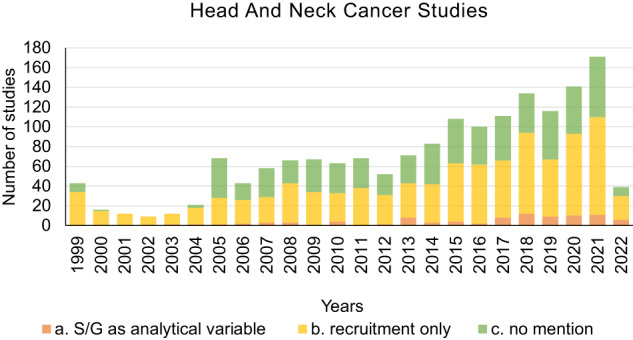
HNC studies submissions on ClinicalTrials.gov. Trend in Head and Neck Cancer (HNC) studies submissions on ClinicalTrials.gov from 1999 to 2022, divided by type of sex/gender mention: recruitment factor, analytical variable or not considered (No mention). Studies were assigned exclusively in one category.

**Fig. 6:**
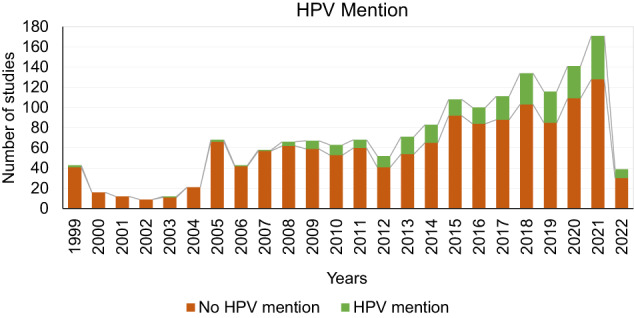
HPV mentioned in HNC studies as of 1999 on ClinicalTrials.gov. Trend regarding HPV mention or no mention in Head and Neck Cancer (HNC) studies submitted on ClinicalTrials.gov from 1999 to 2022. Studies were assigned exclusively in one category.

We analyzed how the mention of HPV varies in HNC studies, and then observed how this mention changed over time. We took into consideration studies in which the role of HPV as a risk factor is well-known, specifically those considering the oral cavity, oropharynx and larynx subsites (Table 3). In subgroup A, studies considering laryngeal-oropharyngeal and oral cavity cancer as at least one condition were selected, whereas subgroup B contains the remaining included studies. There was a significant difference between study status in group A versus B: Group B included more completed studies (*P*-value < 0.001, 49% vs 33%), whereas group A comprised more studies that were not yet recruiting (*P*-value < 0.001, 12% vs 4%). The two groups were significantly equal in the type of study (*P*-value = 0.5). In group A, the number of studies with an eligibility sample size greater than 100 subjects was significantly higher than in group B (P-value < 0.001, 38% vs 26%). The group of studies treating HPV-associated HNC as a condition mentioned significantly more S/G as an analytical variable (*P*-value = 0.03, 7.1% vs 4.4%). Group B included more studies in early phase 1/phase 1, phase 1/phase 2, phase 4 and studies in which the phase was not applicable. No significant difference was observed between the study type and HPV mentioned in the HPV-associated cancers subgroup studies (*P*-value = 0.37, 18% vs 16%). The active studies that were not recruiting yet were those that mentioned HPV significantly more in both subgroups. We then assessed submission of HNC studies to ClinicalTrials.gov from 1999 to April 12, 2022, divided by type of S/G mention (Fig. 5) and HPV mention (Fig. 6). Notably, although HNC studies have increased over time, the consideration of S/G as a relevant variable in the analyses did not follow the same trend. Nevertheless, S/G was taken more into account as an analytical variable in the 2018-2020 timeframe. There was no statistically significant difference upon comparing percentages of S/G mention in 2010 vs 2020 (*P*-value = 1.6% vs 7%). Figure 6 shows the gradual increase in mentioning HPV over time, which becomes particularly evident as of 2008 and onward, reflecting the rise in HPV-positive HNC over the last twenty years^10,13–15^. Therefore, the analysis suggests that while the importance of HPV mention has been correctly noted, S/G perception trails behind.

**Table 3:** Studies characteristics by HPV mention, coded as described in Methods; data were collected on 12 April 2022.

	A. HPV relevant cancer sites					B. Other					A vs B	
	Overall, *N* = 565^a^ (col %)	No mention^b^ *N* = 462^1^	Mention^2^ *N* = 103^a^	OR	*P*-value^c^ CI^d^	Overall, N = 1107^1^ (col %)	No mention^b^ *N* = 926^a^	Mention^b^ *N* = 181^a^	OR	*P*-value^3^ CI^4^	OR	*P*-value^c,d^ CI^e^
*Overall status, (n%)*					<0.001					<0.001		<0.001
Active, not recruiting	56 (9.9)	36 (64)	20 (36)		ref	118 (11)	80 (68)	38 (32)		ref		ref
Completed	187 (33)	164 (88)	23 (12)	0.25	0.12–0.50	537 (49)	495 (92)	42 (7.8)	0.17	0.10–0.29	1.54	1.03–2.31
Not yet recruiting	65 (12)	56 (86)	9 (14)	0.28	0.11–0.70	43 (3.9)	30 (70)	13 (30)	0.91	0.42–1.94	0.67	0.39–1.15
Recruiting/Enrolling by invitation	142 (25)	103 (73)	39 (27)	0.68	0.35–1.31	254 (23)	189 (74)	65 (26)	0.72	0.44–1.16	1.10	0.75–1.62
Unknown status/Suspended	115 (20)	103 (90)	12 (10)	0.20	0.09–0.47	155 (14)	132 (85)	23 (15)	0.36	0.20–0.66	0.80	0.49–1.29
*Study type, (n%)*					0.60					0.7		0.50
Observational/Patient Registry	85 (15)	68 (80)	17 (20)		ref	927 (84)	773 (83)	154 (17)		ref		ref
Interventional	480 (85)	394 (82)	86 (18)	0.87	0.48–1.56	180 (16)	153 (85)	27 (15)	1.12	0.72–1.76	1.14	0.87–1.48
*Enrolment, (n%)*					0.30					0.001		<0.001
≤100^f^	348 (62)	280 (80)	68 (20)	1.26	0.80–1.97	798 (74)	715(85)	118 (15)	0.55	0.39–0.77	0.52	0.42– 0.65
>100	217 (38)	182 (84)	35 (16)		ref	274 (26)	211 (77)	63 (23)		ref		ref
*Sex and gender mention, (n%)*					1.0					0.02		0.030
Analytical variable	40 (7.1)	33 (82)	7 (18)	0.96	0.39–2.35	49 (4.4)	38 (78)	11 (22)	1.98	0.95–4.10	1.79	1.14–2.81
Recruitment only	325 (58)	265 (82)	60 (18)	1.03	0.65–1.62	618 (56)	504 (82)	114 (18)	1.55	1.09–2.19	1.15	0.93– 1.43
No mention	200 (35)	164 (82)	36 (18)		ref	440 (40)	384 (87)	56 (13)				
*Phase, (n%)*					0.32					0.07		<0.001
Early Phase 1 /Phase1	51 (9)	39 (76)	12 (24)		ref	131 (12)	115 (88)	16 (12)		ref		ref
Phase 1/Phase 2	33 (5.8)	29 (88)	4 (12)	0.44	0.13–1.53	68 (6)	52 (76)	16 (24)	2.21	1.02–4.75	1.24	0.73– 2.11
Phase 2/Phase 3	323 (57)	271 (84)	52 (16)	0.62	0.30–1.27	497 (45)	404 (81)	93 (19)	1.65	0.93–2.92	1.66	1.17– 2.37
Phase 4	3 (0.5)	2 (67)	1 (33)	1.62	0.13–19.52	14 (1)	12 (86)	2 (14)	1.19	0.24–5.84	0.55	0.15–1.9
Not Applicable^f^	155 (27.7)	121 (78)	34 (22	0.91	0.43–1.93	397 (36)	343 (86)	54 (14)	1.13	0.62–2.05	1.00	0.69–1.45

Studies were assigned exclusively in one category. Specifically, studies were selected between those mentioning HPV at least once in relevant cancer sites.

^a^*n* (%).

^b^mentioned in the analysis or as eligibility criteria.

^c^Pearson’s Chi-squared test or Fisher’s test.

^d^*P*-value from a Pearson’s Chi-squared test or Fisher’s test for HPV relevant cancer sites versus other studies.

^e^OR with respect to referred category.

^f^missing values included. CI: confidence interval; OR: odds ratio.

For the purpose of our analyses, the mention “pregnant” was found not to be significant because it was not identified in the reported studies.

### Validation analysis

Among the 1672 studies analyzed, 863 had at least one related Pubmed or Embase publication. Of these, 412 mentioned S/G: In 281 studies, S/G was mentioned only upon sample description in the manuscript-related tables, whereas 131 included the S/G variable in univariate and/or multivariate analyses.

The comparison of these published scientific reports with the respective trials reported in ClinicalTrials.gov showed a statistically significant difference in S/G consideration (*P*-value = 0.001) (Supplementary Table 3). 15.2% of the published studies actively considered S/G in the statistical analyses, 8.6% in univariate analysis while 6.6% in multivariate analysis. S/G was more considered in published articles with respect to the reference protocols. Of the 131 publications with S/G variables statistically investigated as univariate or multivariate variable, only 72 mentioned this also in the trial reported in ClinicalTrials.gov.

Supplementary Table 4 and Supplementary Fig. 5 show the S/G mention at the time of study submission on ClinicalTrial.gov (only of the studies published) and S/G mention in the related published manuscripts. Published randomized studies mentioned S/G more compared to published not randomized studies; this difference was not present in the submitted protocols. Studies that dealt with HPV-associated HNC in the protocol mentioned more S/G than the related published articles.

Phase 2/3 studies and studies where phase was not applicable in the protocols mentioned S/G significantly less than early phase 1/ phase 1 studies, but this difference was no longer evident upon manuscript publication. In published manuscripts, S/G mention was higher in studies with a sample size above 100 patients compared with those in which it was below 100 patients.

In conclusion, our analysis revealed that despite the increasing awareness about the importance of S/G in clinical research, the systematic inclusion of S/G as analytical variable in the study design needs to be further implemented, especially in interventional and randomized studies. While HPV infection has been broadly demonstrated to represent a critical factor influencing HNC onset and progression, contributing to significantly improve HNC patient prognosis, S/G perception lags behind.

## Discussion

Men and women with non-sex-related cancers should be considered as biologically distinct patient groups for whom particular treatment methods deserve consideration, especially in diseases or disease subgroups with considerable disparities in epidemiology or outcomes^2^, such as HNC. This is the first systematic review on protocols regarding HNC included in ClinicalTrial.gov and evaluating the importance given to S/G in clinical study design. We focused on S/G as they appear to influence a number of risk factors and behaviors in HNC onset and development. Data are increasingly showing how other sociodemographic variables besides S/G are important factors to account for in cancer, including HNC^12,38,39^. However, the scope of our work was to specifically assess how S/G are taken into consideration.

Indeed, this is a very crucial issue, as the relevance of S/G has been largely debated in the scientific community^4,35,40–50^. The awareness about the importance of always incorporating the S/G variable has also been more recently discussed, highlighting its current importance in many facets of clinical research, including precision oncology^47,48^.

Clearly sex differences in cancer matter, but their translation into clinical oncology is still lagging behind, as lately reviewed^3^.

Our study highlighted that too minimal attention was paid to S/G as a real analytical variable in interventional studies: In 64% of cases (897/1407) it was considered only as an eligibility criterion (Table 1). S/G as an analytical variable was mainly investigated in observational studies, likely due to the known higher risk of bias in observational studies. Therefore, various strategies are implemented to minimize this risk as compared to interventional studies where, instead, randomization already reduces the risk of bias. Furthermore, only trials with a large sample size can investigate S/G as a variable, while observational studies do not necessarily have a sample calculation. We observed that studies with a larger sample size (greater than 100 subjects) mentioned more S/G as an analytical variable, probably because the statistical power allowed for stratified analyses (8% of the studies with more than 100 subjects and 4% with less than 100 subjects). Indeed, a small sample might limit the ability of scientists and clinicians to disaggregate analyses while maintaining statistical power^28^. Clarifying the objectives of a study is critical for sample determination: Observation of phenomena might otherwise be affected by insufficient statistical power to properly address subgroup analyses. Having a large sample could ensure statistical power even for subgroups in which the disease incidence is epidemiologically lower. In particular, in trials, cost and time constraints may adversely affect patient enrolment, so evidence from observational studies would play a major role in formulating hypotheses to be subsequently confirmed by Randomized Controlled Trials (RCTs). Indeed, in observational studies researchers may face challenges even with larger sample size. The smaller number of women could be justified through differences in disease prevalence. However, female patients were shown to be more underrepresented in oncology trials when considering their disease prevalence^25,51,52^. Women are not only under-represented in clinical trials but maybe be also under-treated, as recently reported by Benchetrit et al.^22^. In a large cohort of HNC patients in Northern California, it was found that women were also less likely to receive intensive chemotherapy (35% vs. 46%, *P*-value = 0.006) and radiation (60% vs. 70%, *P*-value = 0.008)^41^. Differences in treatment patterns for women and men with oropharyngeal cancers have also been reported^53^. We are also aware that sex disparities and patient outcomes are still not well delineated, with studies reporting no difference in survival between women and men^41,54–56^.

Most of the studies reviewed by Jagsi et al.^57^ contained a lower percentage of women in relation to the incidence of that type of cancer in the general population, resulting in women being underrepresented in research. A recent cross-sectional study demonstrated persistent female participant underrepresentation in some oncology clinical trials, including HNC^25^. Interestingly, industry-funded trials included proportionally more females compared to all funding sources^25^.

Clinical trials in oncology are usually smaller than in other disciplines and may not be powered for analysis according to S/G. Moreover, in oncology it is important to differentiate controlled trials from other types of interventional research because they allow for a greater level of scientific evidence. Controlled studies compared to uncontrolled studies have lower mention of S/G as an analytical variable (5% and 8%, respectively), with a higher proportion of studies including less than 100 subjects. Significantly greater mention of S/G as an analytical variable was observed in the controlled studies when the sample size was greater than 100 patients. Controlled and uncontrolled studies did not differ in the mention of HPV; however, less than 10% HNC studies that mentioned S/G also mentioned HPV.

Since in ClinicalTrials.gov not all the observational studies could be reported, to overcome the possible protocols underestimation, we analyzed all results on the randomized protocols alone, which are required to be registered on ClinicalTrials.gov. In randomized trials S/G was significantly more mentioned in studies on HPV-associated HNC, with a planned sample of more than 100 patients and in early phase 1 studies versus not applicable. However, we underlined that in the randomized studies, S/G was mentioned as an analysis criterion only in 9% of the trials, 91% mentioned S/G only in terms of eligible population.

We reported a total of 444 active studies, 296 (66.5%) with an expected date of protocol completion from 2019 onwards. They presented the highest proportion of studies including S/G as an analytical variable (7%). We believe that these studies may show greater awareness to the S/G variable because, being more recent, most likely follow the SABV guidelines^35,40^.

In the validations step conducted on the published studies related to the submitted study protocols, the S/G variable is more present in the statistical analyses as variables potentially influencing the study results. Furthermore, in the published studies, the studies that significantly mention the S/G variable were the randomized ones, with a number of patients enrolled greater than 100, Phase 2/3 studies and the ones that do not mention HPV. However, the S/G consideration is low also in these: Out of 863 publications, only 131 included the S/G variable in univariate and/or multivariate analyses.

We observed a higher percentage of S/G as analytical variable in phase 2/3 and interventional studies where the phase is not applicable (such as pilot, feasibility, diagnostic studies).

Since 1994 USA has imposed explicit requirements to include women in clinical trials and to perform sex-based subgroup analysis on study results^58^. Indeed, USA-based medical and research centers showed a greater inclusion of sex as an analytical variable in HNC studies. Over the years the number of HNC studies taking into account the S/G variable has increased, as also recently highlighted^25^, and differences between the sexes have emerged from many studies^15,45,59,60^.

Furthermore, the attention paid to HPV status in HNC studies has been significantly increasing over time and most of the interventional studies that mention HPV were active, not recruiting or not yet recruiting. However, although the mention of HPV in HNC has increased over the past decades when appropriate, S/G is still poorly considered in these cancers. Indeed, only 7 out of 565 studies mention S/G as a relevant variable while also making mention of HPV. Thus, while HPV mention has been correctly recorded, S/G sensitivity straggles.

The contribution of HPV to HNC onset is substantial yet heterogeneous by nation/region, sex, and cancer site, although HPV is predominantly found in the oropharynx subsite^14,61,62^. In 2007, HPV type 16 was recognized as an important risk factor, besides smoking and alcohol consumption, for oropharyngeal squamous cell carcinoma (OPSCC)^63^. Patients with HPV-positive OPSCC have better disease-specific survival than patients with HPV-negative carcinomas^64,65^.

Accordingly, HPV status is now part of the 8^th^ edition TNM classification^66^, as it impacts patients’ prognosis and treatment, while up-to-date sex is not^52^. However, our analysis indicates that S/G variables are generally overlooked in HNC clinical studies, and therefore at least for certain types of HNC, the best evidence could be not sufficient to fully understand the significance of S/G as an independent prognostic factor.

## Limitations and conclusions

Even though ClinicalTrials.com is one of the largest clinical trials registries, there may still be studies missing from the database, especially observational studies, and study information may be incomplete and/or non-detailed. Many researchers do not provide much detail about their analytical plans at the time of study registration and, instead, should be encouraged to do so. It is possible to increase the consideration of S/G in clinical trials and boost the validity of the published results by enforcing the requirement of analytical information and quality control upon registration. To overcome this issue, we validated the sample on the published articles relating to the trials, leading to a more precise analysis of the role of the S/G in the studies but at the same time to a reduction of the samples examined. We think that the issue may be exacerbated by the absence of adequate clarity in the information. Importantly, although S/G are not synonymous, the reported analyses refer to them without distinction. Moreover, there are studies that focus their research on other pathologies, although mentioning the selected keywords. Critiques have been raised against the sharp distinction between S/G because it is not easy to tease out sex as a biological variable and gender as a social category, even more so in clinical studies^67,68^. S/G dimensions continuously interact with one another and in many people traits of masculinity or femininity coexist: More than two thirds of women and men report gender-related characteristics normally attributed to the opposite sex^1,69^. As seen, individual sexual habits play an important role in defining risk and prognosis of oral HPV infection. Nevertheless, their exclusion leads to S/G bias at different levels of health organizations, from daily clinical management, research practices and prevention. These data suggest the need to inform the importance of S/G in future HNC studies and trials. As recently pointed out for COVID-19 studies^28^, a general sex- and gender-sensitive approach should be structurally implemented through mandatory reporting requirements upon registration of clinical trials. This landmark study^28^ can be used to assess how these variables are included in other diseases and cancers. Our findings highlight that S/G biases still persist in different areas of HNC medical practice. Not taking into account S/G can lead to the reproduction of an unequal care system and to a biased knowledge system. Thus, mitigating S/G biases, including a gender approach, will build a more inclusive healthcare system and foster personalized medicine, as medical evidence from clinical and scientific research studies will no longer derive from biased analyses. Finally, there is the need for more articulated guidance on S/G analysis to improve the communication of evidence, inform policy development and guide future research^70^.

## Methods

### Selection of the HNC studies

The main table in the ClinicalTrial.gov (ctgov) schema presented is “studies”. All the studies registered are presented and identified by NCT_ID. The “browse conditions” database in AACT has been populated with Medical Subject Headings keywords (MeSH) published by the National Library of Medicine (NLM), with the goal of better describing studies. In our search related to HNC, we reviewed all conditions investigated by each study on ClinicalTrial.gov, first by looking at all studies that investigated neoplasms by the presence of the following strings: “neopl”, “cancer”, “malignan”, “tumor”, “carcino”, “onco”. Secondly, by examining which studies involved relevant regions of the head-neck: “head”, “neck”, “mouth”, “oral cavity”, “pharynx”, “larynx”, “nose”, “paranasal”, “salivary”, “uadt”, “upper aerodigestive tract”, “gingiva”, “otorinolar”, “tongue”, “tonsil”. We constructed an index for the number of conditions concerning HNC out of the total number of conditions examined by each study. Only studies that treated HNC as a unique investigated condition were included in the analysis. To search for terms related to S/G we targeted the following registration fields: the official and brief trial titles (studies), description (detailed description), description (brief summaries), measure and description (design outcomes), title (design groups), description (intervention) and population and criteria (eligibilities). To verify the inclusion of all studies registered in The National Clinical Trial system, a systematic review was conducted on PubMed and Embase. The results are shown in Supplementary Fig. 1 and Supplementary Fig. 2. We decided to consider only protocols registries on ClinicalTrial.gov, which is considered the largest universally accepted registry of clinical trials and recognized by the FDA^71^. The review was carried out double blinded by two independent researchers (AG and SG) to ensure objective evaluation. No internationally registered studies were found to be missing on ClinicalTrial.gov. The search for mention of HPV took place in all fields by searching for the following strings: “human papillomavirus”, “hpv human papillomavirus”,“alphapapillomaviruses”, “hpv human papillomaviruses”, “papillomavirus, human”, “human papillomavirus”, “hpv”, “human papillomaviruses, hpv”, “papillomaviruses, human”, “human papillomaviruses”. The selection of the strings to search for was done by observing MeSH terms. We removed studies listed as ‘Withdrawn’ or ‘Terminated’, and studies designed to include a single sex. Additional analyses were performed on the controlled and uncontrolled studies, randomized and non-randomized studies. The arms’ description was investigated within the dataset design groups. If at least one arm was defined as “Experimental”, “Active Comparator”, “Placebo Comparator”, “Sham Comparator” the study was classified as controlled, otherwise it was classified as uncontrolled (“No intervention”, “Other”).

The condition “Study design, Allocation” was used to classify the protocols as randomized or non-randomized.

More detailed information on the meaning of the terminology used is available at https://clinicaltrials.gov/ and at https://aact.ctti-clinicaltrials.org/data_dictionary.

### Identification of attention given to S/G and HPV

Scientists are required by ClinicalTrials.gov to declare eligible sexes as a criterion by selecting an option from the predefined list (“All”, “Male” or “Female”). We used these data elements to remove single-sex study from our analysis. For studies with eligibility open to ‘All’ sexes, we identified S/G by searching for the following terms: “sex”, “gender”, “woman”, “women”, “man”, “men”, “female”, “females”, “male”, “males”, “girl”, “girls”, “boy”, “boys”, “pregnan” and “transg”. We observed the presence of sex-related terms in the official and brief trial titles, studies’ detailed description, brief summaries description, design outcomes, design groups and intervention description, as well as the mention of sex as an eligibility criterion.

All studies that mentioned terms related to sex or gender were examined and assigned to one of the following categories depending on the mention of one or more of the terms listed within the sections given for each study: (a) S/G mentioned as an analysis criterion (section: detailed description, brief description, brief title, official title, outcome measure, design description, groups title, interventions description), (b) studies with only mention of sex/gender terms in the eligible population (section: gender population and gender criteria) and (c) no mention at all (in all the sections considered by this study). A sub-analysis was performed concerning the mention of HPV-related oral cavity, oropharyngeal and laryngeal cancers [“oral cavity”, “pharyn”, “laryn”, “tonsil”, “base of tongue”].

For the validation step, the trials published in scientific journals that mentioned terms related to sex or gender were examined and assigned to one of the following categories: (a) published studied including S/G as variables in univariate or multivariate analysis, (b) S/G reported as population characteristics, (c) S/G never described. Categorical variables were summarized with frequencies and percentages. Differences between groups were tested using Pearson’s chi-square test or Fisher’s test for categorical variables. The S/G mention was evaluated as a comparison between mention (considered as (a) and (b)) versus not mention (c) using the estimated odds ratio with a 95% confidence interval (CI).

### Ethics statement

The European Institute of Oncology approved the study. Participants’ consent was waived as the data were publicly available, so there was no need to seek consent.

### Reporting summary

Further information on research design is available in the Nature Research Reporting Summary linked to this article.

### Supplementary information

Supplemental Material

Reporting Summary

## Data Availability

The datasets generated and/or analyzed during the current study are available from the corresponding author on reasonable request. Data were obtained through a query of the relational database AACT “Aggregate Analysis of ClinicalTrials.gov” on 12 April 2022; AACT contains all publicly available ClinicalTrials.gov data; the access was made through R software. We considered the studies included in ClinicalTrial.gov and did a search to assess whether other protocols not included in this database were published (PubMed and Embase). For the validation step, two different MDs authors (MT and RDB) independently verified which studies registered on ClinicalTrials.gov had publications indexed on PubMed and/or Embase to verify the study results in the publication set.

## Code availability

All the codes and guidance for R scripts can be found on GitHub: https://github.com/auroragaeta/sex-and-gender-hnc.
