# Supplementary material for: Are sex and gender considered in head and neck cancer clinical studies?
Source: NPJ Precis Oncol. 2023 Sep 7;7:84. doi: 10.1038/s41698-023-00439-z (PMC10484986; doi:10.1038/s41698-023-00439-z)
Supplement: Supplementary file 1 — Supplemental Material [file 41698_2023_439_MOESM1_ESM.pdf]

Supplementary Figure 1. PubMed search

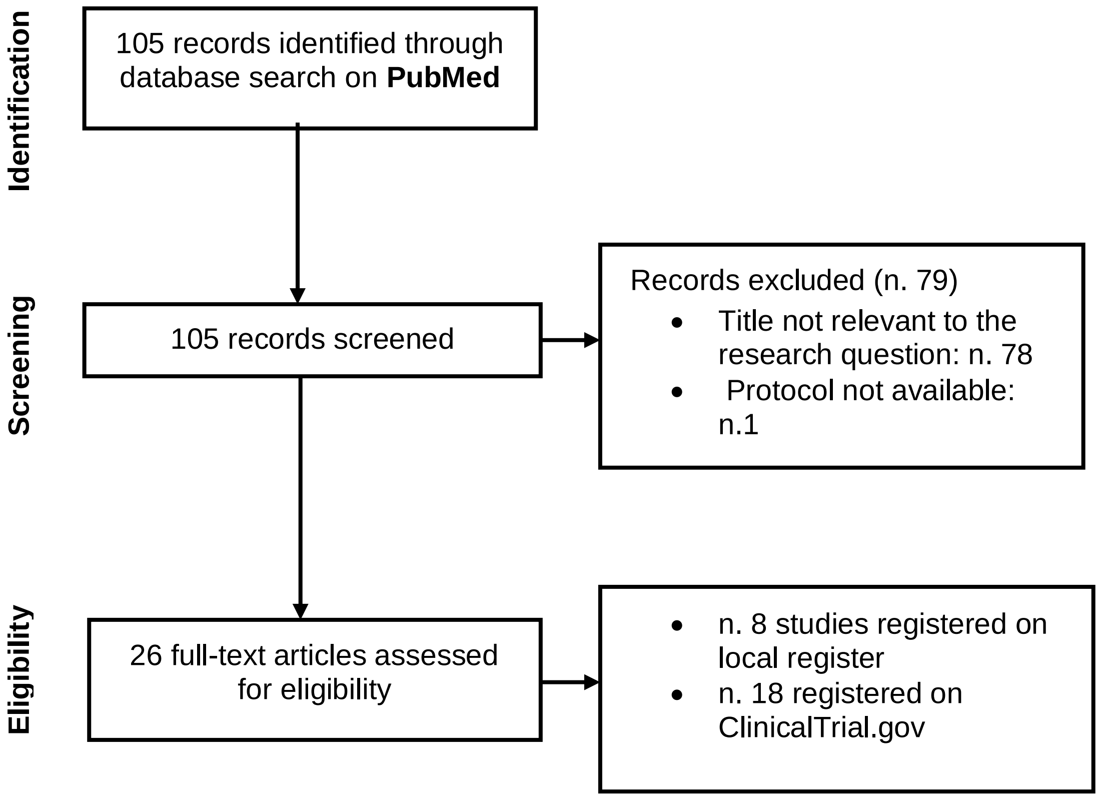

Supplementary Figure 1. ("study protocol*" OR "research protocol*" OR "trial protocol*") AND (((neoplasms [MeSH Terms] OR cancer* [TW] OR neoplas*[TW] OR carcinoma*[TW] OR maligna*[TW] OR tumor*[TW] OR tumour*[TW] OR oncolog*[TI]) AND ((head OR neck OR mouth OR "oral cavity" OR pharynx OR pharyngeal* OR larynx OR nose OR nasal OR paranasal OR salivary OR uadt OR "upper aerodigestive" OR "upper aero-digestive" OR gingival OR otorhinolar* OR "oto rhino laryng*" OR "otorhino laryng*" OR tongue OR tonsil* or palatal))) OR ("Head and Neck Neoplasms"[Mesh]))

Article (Books and documents, clinical trial, Randomized Controlled Trial, with full text available)

Supplementary Figure 2. Embase search

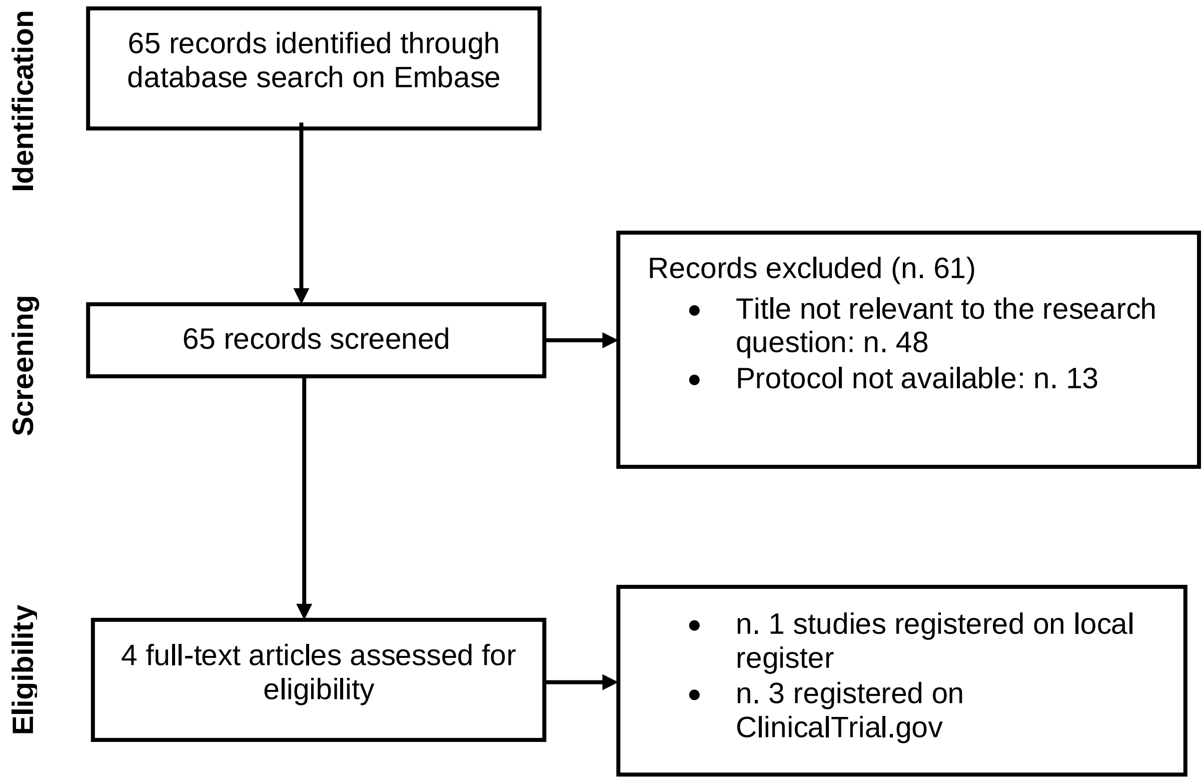

Supplementary Figure 2. ('study protocol*' OR 'research protocol*' OR 'trial protocol*') AND ('head and neck tumor'/exp OR 'head and neck tumor' OR ((cancer*:ti OR neoplas*:ti OR carcinoma:ti OR maligna*:ti OR tumor:ti OR tumour* OR oncolog*:ti) AND ('head' OR 'head'/exp OR head OR 'neck' OR 'neck'/exp OR neck OR 'mouth' OR 'mouth'/exp OR mouth OR 'oral cavity'/exp OR 'oral cavity' OR 'pharynx' OR 'pharynx'/exp OR pharynx OR pharyngeal* OR 'larynx' OR 'larynx'/exp OR larynx OR 'nose' OR 'nose'/exp OR nose OR nasal OR paranasal OR salivary OR uadt OR 'upper aerodigestive' OR 'upper aero-digestive' OR gingival OR otorhinolar* OR 'oto rhino laryng*' OR 'otorhino laryng*' OR 'tongue' OR 'tongue'/exp OR tongue OR tonsil* OR palatal)) OR 'upper aerodigestive tract cancer'/exp OR 'upper aerodigestive tract cancer') AND [embase]/lim NOT ([embase]/lim AND [medline]/lim) AND ('article'/it OR 'review'/it)

Supplementary Figure 3

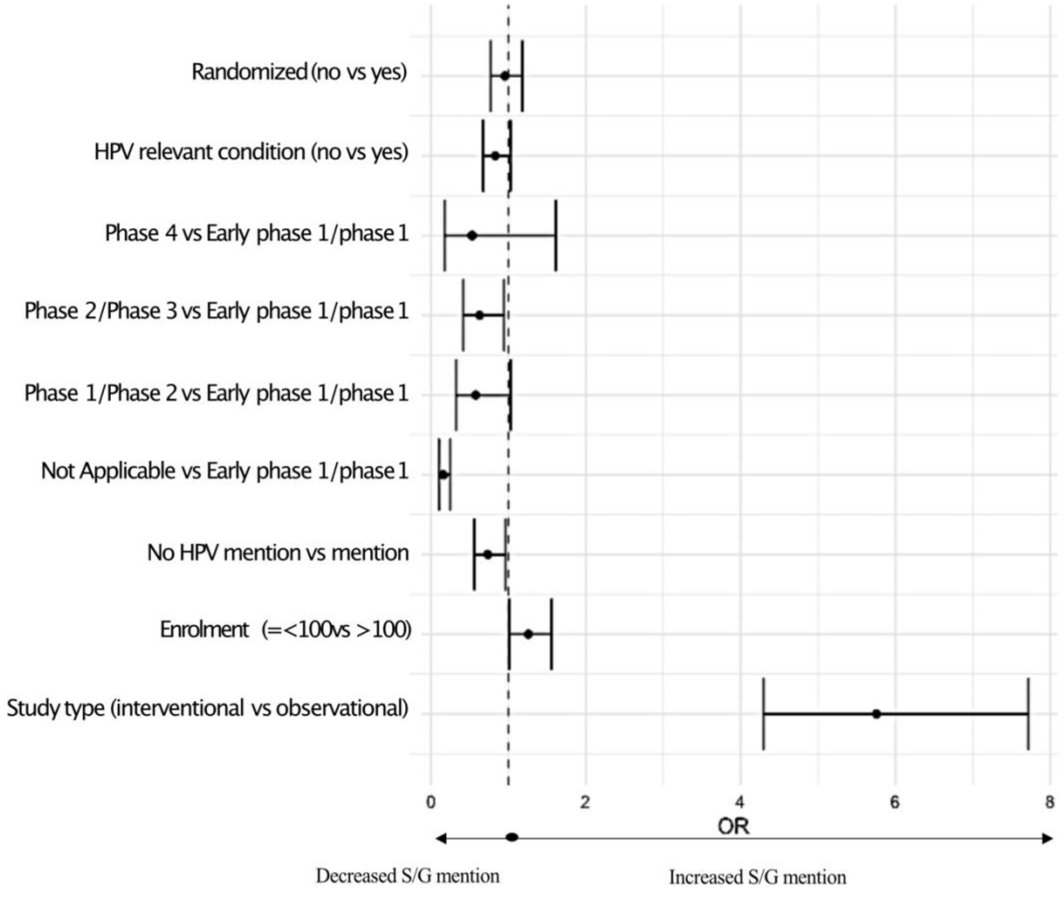

Supplementary Figure 3. Graphical representation of the Odds Ratios (OR) and confidence interval relative to the S/G mention in the overall sample. The estimated OR is represented by the dot, while lines represent the 95%CI. The 95%CI fully to the left/right of the vertical axis, correspondent to OR=1, means a significant lower/higher S/G mention in the category of interest.

Supplementary Figure 4

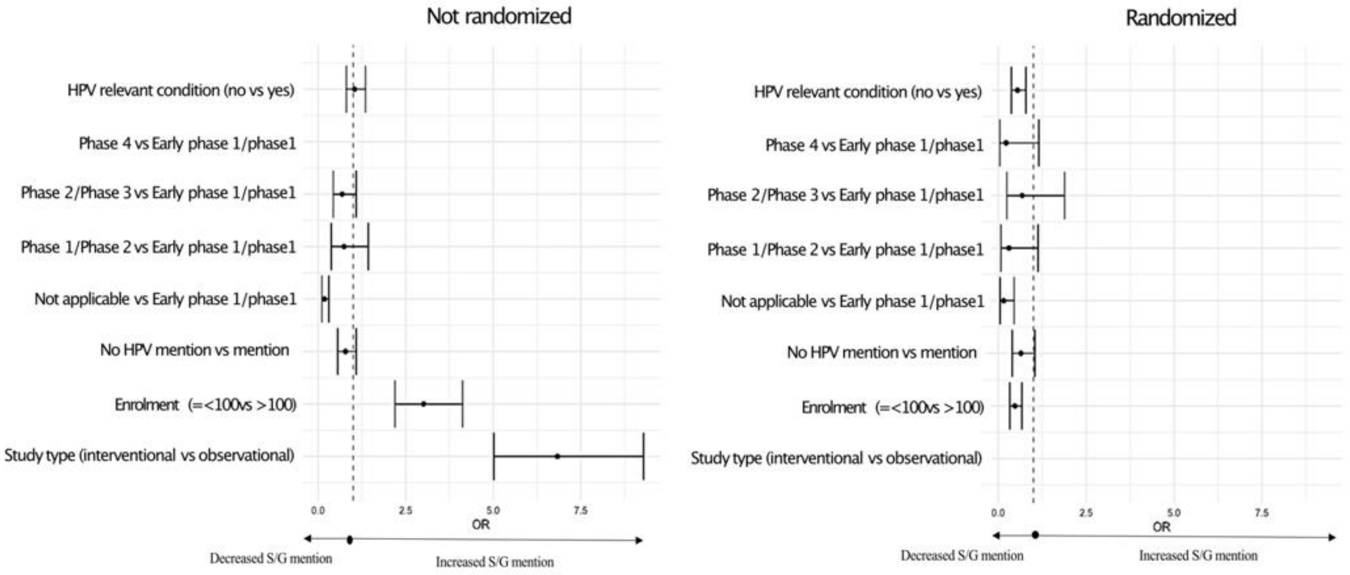

Supplementary Figure 4. Graphical representation of the Odds Ratios (OR) and confidence interval relative to the S/G mention by randomization. The estimated OR is represented by the dot, while lines represent the 95%CI. The 95%CI fully to the left/right of the vertical axis, correspondent to OR=1, means a significant lower/higher S/G mention in the category of interest.

Supplementary Figure 5

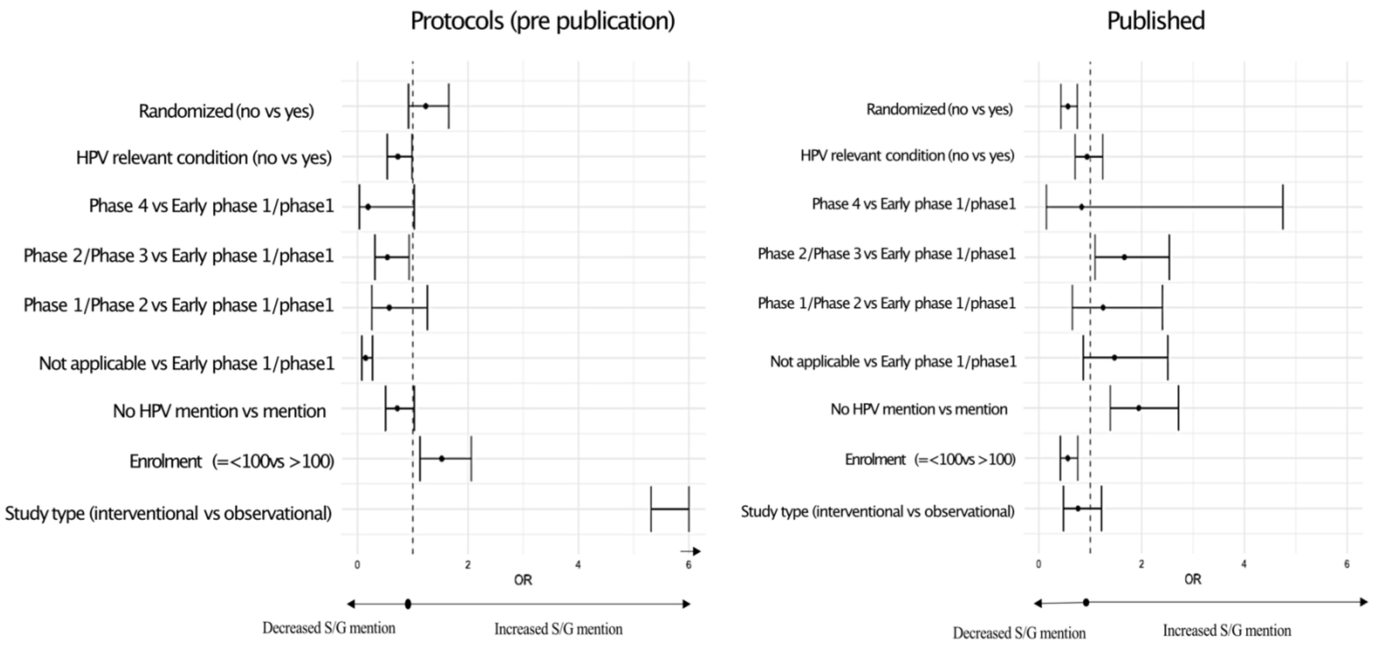

Supplementary Figure 5. Graphical representation of the Odds Ratios and confidence interval relative to the S/G mention of the protocols analyzed and of the published studies. The estimated OR is represented by the dot, while lines represent the 95%CI. The 95%CI fully to the left/right of the vertical axis, correspondent to OR=1, means a significant lower/higher S/G mention in the category of interest.

Supplementary Table 1

**Supplementary Table 1/a.** Sex/Gender mention in ClinicalTrial.gov by study characteristics			
**Characteristic**	S/G mentioned[1] N=1,032[2]	S/G NO mention[1] N=640[2]	P-value[3]
**Mention in ClinicalTrial.gov(extended)**			-
S/G mentioned[1]	89	-	
only eligibility	943	-	
no mention	-	640	
**Study type**			<0.001
Interventional	960 (68)	447 (32)	
Observational/Patient Registry	72 (27)	193 (73)	
**Overall status**			<0.001
Active, not recruiting	125 (72)	49 (28)	
Completed	415 (57)	309 (43)	
Recruiting/enrolling by invitation	265 (67)	132 (33)	
Not yet recruiting	73 (68)	34 (32)	
Unknown status/Suspended	154 (57)	116 (43)	
**Enrolment**			0.037
≤100[4]	750 (63,5)	431 (36,5)	
>100	282 (57)	209 (43)	
**HPV mention**			0.025
No Mention	840 (61)	548 (39)	
Mention	192 (68)	92 (32)	
**Studies with a relevant HPV role**			0.084
Other	667 (60)	440 (40)	
Oral cavity, oropharynx and larynx subsites	365 (65)	200 (35)	
**Phase**			<0.001
Early Phase 1/Phase1	149 (82)	33 (18)	
Phase 1/Phase 2	73 (72)	28 (28)	
Phase 2/Phase 3	606 (74)	214 (26)	
Phase 4	12 (71)	5 (29)	
Not Applicable[4]	192 (35)	356(65)	
**Randomized**			0.7
No	696 (61)	438 (39)	
Yes	336 (62)	202 (38)	

[1] Sex/gender mentioned in the analysis or as eligibility criteria, [2] n (%), [3] Pearson's Chi-squared test,
[4] missing values included

Supplementary Table 1/b. Sex/Gender mention in controlled studies and uncontrolled studies, Odds ratio of any Sex/Gender mention vs no Sex/Gender mention

	Controlled studies					Uncontrolled studies					Controlled vs uncontrolled	
**Characteristic**	**Overall N=1,212[1] (col %)**	**S/G mentioned[2] N=843[1]**	**S/G No mention[2] N=369[1]**	**OR**	**P-value[3] CI[4]**	**Overall N=204 (col %)**	**S/G mentioned[2] N=20[1]**	**S/G No mention[2] N=184[1]**	**OR**	**P-value[3] CI[4]**	**OR**	**P-value[3] CI[4]**
**Sex and gender mention, (n%)**					-					-		**<0.000**
Analytical variable		58(5)	-				20(10)	-				ref
Recruitment only		785(65)	-				43(21)	-			6.29	3.47-11.39
No mention		-	369 (30)				-	141(69)			0.90	0.52-1.55
**Study type (n%)**					-					**0.016**		-
Interventional	**1,212**	843 (70)	369 (30)			**52 (25)**	23 (44)	29 (56)	2.22	1.15-4.27		
Observational/ Patient Registry						**152 (75)**	40 (26)	112 (74)		ref		
**Overall status (n%)**					**0.003**					**0.95**		**0.07**
Active, not recruiting	**149 (12)**	116 (78)	33 (22)		ref	**21 (10)**	7(33)	14 (67)		ref		ref
Completed	**481 (40)**	310 (64)	171 (36	0.51	0.33-0.79	**81 (40)**	25 (31)	56 (69)	0.89	0.32-2.48	0.83	0.50-1.39
Not yet recruiting	**95 (8)**	70 (74)	25 (26)	0.79	0.43-1.44	**9 (4)**	3 (33)	6 (67)	1	0.19-5.24	1.48	0.65-3.38
Recruiting/Enrolling by invitation	**318 (26)**	236 (74)	82 (26)	0.81	0.51-1.29	**51 (25)**	17 (33)	34 (67)	1	0.34-2.93	0.87	0.50-1.51
Unknown status/Suspended	**169 (14)**	111 (66)	58 (34)	0.54	0.33-0.89	**42 (21)**	11 (26)	31 (74)	0.70	0.22-2.21	0.56	0.32-1.00
**Enrolment (n%)**					**0.372**					**0.012**		**<0.000**
≤100[5]	**879 (73)**	605 (69)	274 (31)	0.88	0.66-1.16	**115 (56)**	44 (38)	73 (62)	2.21	1.18-4.17	2.0	1.47-2.71
>100	**333 (27)**	238 (71)	95 (29)		ref	**88 (43)**	8 (9.1)	69 (78)		ref		ref
**HPV mention (n%)**	**222 (18)**	13 (5.9)	49 (22)	1.68	**0.003**	**43 (21)**	11 (26)	32 (74)	0.72	**0.397**	**0.83**	**0.349**

					1.19-2.37					0.33-1.54		0.58-1.21
**Phase (n%)**					**<0.000**					**0.012**		**<0.000**
Early Phase 1/Phase1	**159 (13)**	131 (82)	28 (18)		ref	**4 (2)**	3 (75)	1 (25)		ref		ref
Phase 1/Phase 2	**85 (7)**	65 (76)	20 (24)	0.16	0.10-0.26	**2 (1)**	1 (50)	1 (50)	0.33	0.00-11.93	1.06	0.19-5.95
Phase 2/Phase 3	**717 (59)**	533 (74)	184 (26)	0.69	0.36-1.32	**15 (7)**	9 (60)	6 (40)	0.50	0.04-6.01	1.20	0.39-3.67
Phase 4	**16 (1)**	12 (75)	4 (25)	0.61	0.39-0.96		-	-		-		-
Not Applicable[5]	**235 (19)**	102(43)	133 (57)	0.16	0.10-0.26	**183 (90)**	50 (27)	133 (73)	0.12	0.01-1.23	0.03	0.01-0.08

[1] n (%),[2] sex/gender mentioned in the analysis or as eligibility criteria, [3] Pearson's Chi-squared test, [4] OR with respect to referred category, [5] missing values included
CI: confidence interval; OR: odds ratio

Supplementary Table 2

**Supplementary Table 2.** Planned Sex/Gender mention in ClinicalTrial.gov by randomization				
	**Not randomized**, N=1134		**Randomized**, N=538	
	**S/G Mention**[1] N=696[2]	**S/G No Mention**[1] N=438[2]	**S/G Mention**[1] N=336[2]	**S/G No Mention**[1] N=202[2]
**S/G mention in ClinicalTrial**[1]				
S/G mentioned[1]	58 (8.3)	-	31 (9.2)	-
Only eligibility	638 (92)	-	305 (91)	-
No mention	-	438	0 (0)	202
**Study type**				
Interventional	624 (90)	245 (56)	336	202
Observational / Patient Registry	72 (10)	193 (44)	0 (0)	0 (0)
**Overall status**				
Active, not recruiting	78 (11)	29 (6.6)	47 (14)	20 (9.9)
Completed	316 (45)	209 (48)	99 (29)	100 (50)
Recruiting/enrolling by inv	167 (24)	98 (22)	98 (29)	34 (17)
Not yet recruiting	43 (6.2)	26 (5.9)	30 (8.9)	8 (4.0)
Unknown status/Suspended	92 (13)	76 (17)	62 (18)	40 (20)
**Enrollment**				
≤100[3]	592 (88)	310 (72)	129 (39)	115 (57)
>100	78 (12)	123 (28)	204 (61)	86 (43)
**HPV mention**				
No Mention	571 (82)	374 (85)	269 (80)	174 (86)
Mention	125 (18)	64 (15)	67 (20)	28 (14)
**Studies with a relevant HPV role**				
Other	489 (70)	304 (69)	178 (53)	136 (67)
Oral cavity, oropharynx and larynx subsites	207 (30)	134 (31)	158 (47)	66 (33)
**Phase**				
Early Phase 1/Phase1	131 (21)	28 (11)	18 (5.4)	5 (2.5)
Phase 1/Phase 2	62 (9.9)	18 (7.3)	11 (3.3)	10 (5.0)
Phase 2/Phase 3	346 (55)	108 (44)	260 (77)	106 (52)
Phase 4	8 (1.3)	0 (0)	4 (1.2)	5 (2.5)
Not Applicable[3]	77 (12)	91 (37)	43 (13)	76 (38)
**S/G mention in published manuscripts**				
S/G never described	226 (32)	91 (21)	89 (26)	45 (22)
Reported	130 (19)	59 (13)	55 (16)	37 (18)
Univariate	13 (1.9)	15 (3.4)	25 (7.4)	21 (10)
Multivariate	7 (1.0)	12 (2.7)	27 (8.0)	11 (5.4)
No pubblication	320 (46)	261 (60)	140 (42)	88 (44)
**Randomized**				
No	696	438		
Yes			336	202

[1] n (%)

Supplementary Table 3

Supplementary Table 3. Publications on Pubmed and/or Embase related to each study registered on ClinicalTrial.gov

		Type of mention in ClinicalTrial.gov		
**Characteristic**	**Overall N=863**[1]	**S/G mentioned or mentioned as eligibility criteria**[2] **N=572**[1]	**S/G No mention N=291**[1]	**P-value**[3]
**Type of S/G mention in the final manuscripts**[2]				0.01
**S/G never described**	451 (52%)	315 (55%)	136 (47%)	
**Reported**	281 (33%)	185 (32%)	96 (33%)	
**Univariate**	74 (8.6%)	38 (6.6%)	36 (12%)	
**Multivariate**	57 (6.6%)	34 (5.9%)	23 (7.9%)	
**Not published**	809	460	349	

[1] n (%), [2] sex/gender mentioned in the analysis or as eligibility criteria, [3] Pearson's Chi-squared test

Supplementary Table 4

Supplementary Table 4. Sex/Gender mention in protocols (only published) and S/G mention in manuscripts published

	S/G mention	In protocol (only published)		In published paper	
**Characteristic**	**Overall N (%)863**[1]	**S/G Mention**[2] N=572[1]	**S/G No mention**[2] N=291[1]	**S/G Mention**[2] N=412[1]	**S/G No mention**[2] N=451[1]
**S/G mention in ClinicalTrial**[2]					
S/G mentioned	**36**	36	0 (0)	16 (44)	20 (56)
Only eligibility	**536**	536	0 (0)	241 (45)	295 (55)
No mention	**291**	0 (0)	291	155 (53)	136 (47)
**Study type**					
Interventional	**785**	556 (71)	229 (29)	370 (47)	415 (53)
Observational / Patient Registry	**78**	16 (21)	62 (79)	42 (54)	36 (46)
**Enrollment**					
≤100[3]	**592**	411 (69)	181 (31)	258 (43)	334 (57)
>100	**271**	161 (59)	110 (41)	154 (57)	117 (43)
**HPV mention**					
No Mention	**675**	437 (65)	238 (35)	346 (51)	329 (49)
Mention	**188**	135 (72)	53 (28)	66 (35)	122 (65)
**HPV as relevant condition**					
Other	**570**	371 (65)	199 (35)	269 (47)	301 (53)
Oral cavity, oropharynx and larynx subsites	**293**	201 (69)	92 (31)	143 (49)	150 (51)
**Phase**					
Early Phase 1/Phase1	**112**	94 (84)	18 (16)	42 (38)	70 (62)
Phase 1/Phase 2	**56**	42 (75)	14 (25)	24 (43)	32 (57)
Phase 2/Phase 3	**498**	368 (74)	130 (26)	249 (50)	249 (50)
Phase 4	**6**	3 (50)	3 (50)	2 (33)	4 (67)
Not Applicable[3]	**191**	65 (34)	126 (66)	95(50)	96 (50)
**Randomized**					
No	**553**	376 (68)	177 (32)	236 (43)	317 (57)
Yes	**310**	196 (63)	114 (37)	176 (57)	134 (43)
**S/G mention in published manuscripts**					
S/G never described	**451**	315 (70)	136 (30)	0 (0)	451
Reported	**281**	185 (66)	96 (34)	281	0 (0)
Univariate	**74**	38 (51)	36 (49)	74	0 (0)
Multivariate	**57**	34 (60)	23 (40)	57	0 (0)

[1] n (%), [2] sex/gender mentioned in the analysis or as eligibility criteria, [3] missing values included
